# SALC-Net: A lightweight contour-preserving segmentation network for yak body segmentation in complex grazing environments

**DOI:** 10.1371/journal.pone.0353672

**Published:** 2026-08-04

**Authors:** Wang Zhang, Changqi Fu, Jiayi Xing, Shilei Xing, Dedong Gao, Qiangqiang Yao

**Affiliations:** 1 School of Mechanical Engineering, Qinghai University, Xining, China; 2 School of Animal Husbandry and Veterinary Medicine, Qinghai University, Xining, China; University of Electronic Science and Technology of China, CHINA

## Abstract

Accurate livestock segmentation is a key prerequisite for non-contact image-based analysis and intelligent pasture management, yet remains challenging on resource-constrained edge devices. Although lightweight networks are suitable for real-time deployment, they often suffer from limited geometric adaptability and insufficient boundary preservation, which reduces the reliability of downstream shape-related analysis for non-rigid livestock targets. To address this issue, we propose SALC-Net, a lightweight segmentation framework for yak body contour extraction. SALC-Net combines a re-parameterized MobileNetV2 backbone for efficient inference, a Scale-Adaptive Efficient Dynamic Pyramid (SA-EDP) module for low-cost adaptive receptive-field modeling, and a Linear Cross-Scale Fusion (LCSF) module for contour-preserving feature reconstruction. Experiments on a custom high-altitude yak dataset show that SALC-Net achieves 93.37% mIoU at 129 FPS, demonstrating a favorable trade-off between segmentation accuracy and real-time efficiency.

## 1 Introduction

The yak is an important indigenous livestock species distributed across the Qinghai–Tibet Plateau and surrounding alpine regions. It has long adapted to extreme environments characterized by hypoxia, low temperature, intense ultraviolet radiation, and seasonal forage fluctuation. As a major source of meat, milk, draft power, and household income in high-altitude pastoral areas, the yak plays an essential role in regional food security and pastoral economic development [1]. Previous studies have shown that yaks have evolved integrated adaptations involving the respiratory and circulatory systems, energy metabolism, digestive efficiency, and genetic mechanisms [2]. In addition, research on genomic adaptation and sustainable production systems has highlighted the importance of improving production efficiency, protecting ecological security, and promoting smart farming in yak husbandry [3]. Therefore, precise image-based measurement, body measurement, body weight estimation, and behavior monitoring are regarded as key components of intelligent yak farming.

With the development of computer vision, deep learning, and intelligent sensing, image- and video-based non-contact measurement has become an important approach in precision livestock farming. Visual data from two-dimensional images, depth images, and three-dimensional point clouds have been widely used for body measurement extraction, body weight prediction, conformation assessment, and behavior recognition in livestock, with advantages in measurement efficiency, reduced human disturbance, and continuous monitoring [4–7].

Substantial progress has been made in body weight estimation, body measurement, and behavior analysis. For example, cloud–edge collaborative methods and YOLO-based frameworks have been applied to yak body weight estimation under practical conditions [8]. In yaks, YOLO-based body-parameter detection has been used for live body weight estimation [9]. In cattle, semantic segmentation, keypoint detection, stereo vision, and point-cloud-based approaches have been applied to body measurement and weight prediction, demonstrating the effectiveness of non-contact visual perception for livestock phenotyping [10–16]. In addition, face recognition, pose estimation, and skeleton-based behavior classification have been introduced for yak feeding monitoring, behavior recognition, and posture analysis in complex environments [17–19]. At the methodological level, recent segmentation architectures, including efficient Transformer-based networks, encoder-decoder models, pyramid parsing networks, high-resolution representations, promptable foundation models, real-time segmentation networks, and point-cloud segmentation methods, have provided an important technical basis for livestock segmentation, measurement, and visual phenotyping [20–28,29–31]. Meanwhile, yak-related phenotypic studies have increasingly adopted deep learning for body weight estimation, feeding behavior monitoring, behavior classification, pose estimation, and non-contact body measurement, indicating the practical need for robust visual perception methods in yak husbandry [8,9,17–19,32].

Despite these advances, dedicated studies on yaks remain limited. Compared with dairy or beef cattle, yaks are usually raised in more challenging grazing environments and often present dark coat color, weak surface texture, large posture variation, cluttered backgrounds, illumination changes, and severe mutual occlusion. These characteristics increase the difficulty of target detection, semantic segmentation, keypoint localization, and parameter extraction. Moreover, many existing methods perform well under controlled conditions but remain limited in lightweight deployment, cross-scene generalization, real-time inference, and robustness in natural pasture environments.

To address these challenges, a Scale-Adaptive Lightweight Context Network (SALC-Net) is proposed for contour-sensitive yak segmentation under lightweight deployment constraints. A re-parameterized MobileNetV2 backbone is adopted to improve the balance between representation capacity and inference efficiency. A Scale-Adaptive Efficient Dynamic Pyramid (SA-EDP) module is introduced for low-cost adaptive receptive-field modeling to better handle scale variation, pose diversity, and local deformation. In addition, a Linear Cross-Scale Fusion (LCSF) module is designed to recover fine spatial details lost during downsampling and to enhance contour-preserving reconstruction.

The main contributions of this work are summarized as follows:

1. A lightweight yak segmentation framework, termed SALC-Net, is proposed to improve the balance between contour-preserving segmentation accuracy and deployment efficiency in pastoral edge-computing scenarios.
2. A Scale-Adaptive Efficient Dynamic Pyramid (SA-EDP) module is introduced to achieve low-cost adaptive receptive-field modeling for scale variation, pose diversity, and local deformation.
3. A Linear Cross-Scale Fusion (LCSF) module is designed to enhance contour-preserving reconstruction through semantic-guided cross-scale feature fusion under lightweight decoding constraints.

## 2 Related work

### 2.1 Lightweight semantic segmentation

Lightweight semantic segmentation has become an important research direction for resource-constrained scenarios. MobileNetV2 introduced inverted residual and linear bottleneck structures, significantly reducing model complexity while maintaining a favorable balance between efficiency and accuracy [33]. BiSeNet further decoupled spatial-detail preservation and contextual representation through a bilateral network structure, improving real-time segmentation efficiency [29]. PIDNet incorporated the idea of proportional-integral-derivative control into segmentation network design, achieving a good trade-off between accuracy and inference speed [30]. These studies indicate that lightweight segmentation depends not only on parameter reduction, but also on effective preservation of spatial details and high-level semantic information under limited computational budgets.

### 2.2 Multi-scale contextual modeling and boundary preservation

In complex natural scenes, segmentation performance is strongly influenced by multi-scale contextual modeling and boundary-detail recovery. SegFormer introduced a hierarchical Transformer encoder and lightweight MLP decoder to aggregate multi-level features efficiently [20]. Encoder-decoder and context aggregation models, such as SegNet, TransUNet, DeepLabV3 + , PSPNet, and HRNet, further demonstrate the importance of spatial reconstruction, long-range dependency modeling, atrous convolution, pyramid parsing, and high-resolution representation for dense prediction tasks [21–25]. Point-cloud segmentation and adaptive cutting-plane recognition methods have also supported non-contact livestock body measurement in three-dimensional phenotyping scenarios [26]. More recent segmentation benchmarks and foundation models, including Segment Anything and RTFormer, have further advanced promptable mask generation and real-time Transformer-based segmentation [27,28]. In addition, boundary-aware and contour-preserving methods have shown that explicit boundary modeling and pseudo-boundary mitigation can improve segmentation consistency in complex scenes [31,34]. These studies suggest that, for animal segmentation tasks targeting body-shape analysis, region-level accuracy alone is insufficient; contour-sensitive detail recovery and adaptive scale modeling are also essential.

### 2.3 Livestock visual segmentation

In livestock vision, segmentation research has expanded from generic object scenes to individual identification and body-region extraction in cattle, sheep, and other farm animals. Feng et al.[35] improved DeepLabV3+ for cattle-body segmentation in complex farming environments, enhancing target extraction under complicated backgrounds. Zhao et al.[36] proposed an instance segmentation method for sheep images, addressing adhesion, irregular boundaries, and overlapping occlusion. Recent yak phenotyping studies have also shown the value of deep learning-based visual perception for non-contact body measurement and posture-related parameter extraction [32]. Although these studies have demonstrated the potential of deep learning methods in livestock vision, most existing work has focused on detection, keypoint localization, or body-region extraction, while lightweight contour-sensitive segmentation directly supporting quantitative yak morphology analysis remains limited.

## 3 Materials and methods

### 3.1 Datasets

A total of 1,550 high-resolution images (≥1920 × 1080 pixels) were collected from four pastoral regions in Qinghai Province at elevations of 2,800–4,500 m. The dataset covers four challenging scenarios: complex backgrounds (35%), occlusion and multiple targets (25%), pose variation (20%), and illumination changes (20%). All images were manually annotated using LabelMe with approximately 120 control points per yak to ensure contour fidelity. The dataset was partitioned into training (1,240 images) and validation (310 images) sets using an 80:20 split with stratified sampling to maintain consistent scenario distribution across both subsets. Given the dataset size and scenario diversity, a two-way split was adopted to ensure sufficient samples for stable performance assessment. The validation set was additionally used as a held-out test set for performance evaluation.

### 3.2 A scale-adaptive lightweight context network for efficient yak segmentation

Precise segmentation of plateau yaks in unstructured grazing environments is challenged by three factors: scale variation, insufficient cross-scale feature interaction, and boundary degradation caused by complex backgrounds and occlusion. Existing lightweight segmentation networks often rely on fixed receptive fields and simple feature fusion strategies, which are computationally efficient but may be inadequate for preserving geometric detail in non-rigid livestock targets.

To address these limitations, we propose SALC-Net, a lightweight segmentation framework built on MobileNetV2. The key novel contributions lie in the Scale-Adaptive Efficient Dynamic Pyramid (SA-EDP) module and the Linear Cross-Scale Fusion (LCSF) module, which together enable high segmentation accuracy while maintaining low computational cost for edge deployment.

**Fig 1:**
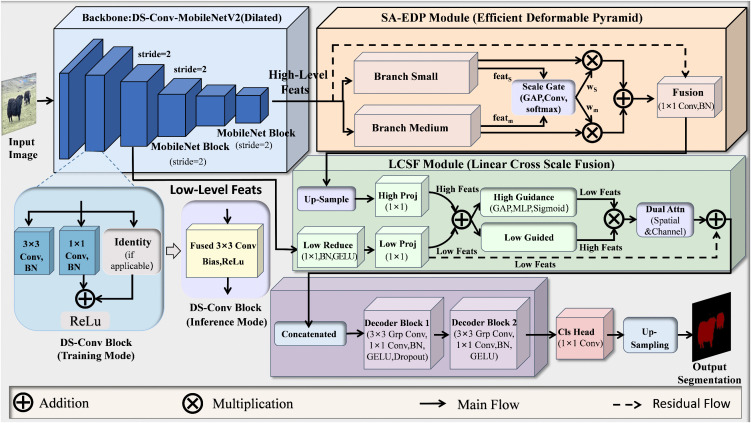
Architecture of the proposed SALC-Net for plateau yak semantic segmentation.

SA-EDP introduces dynamic, spatially-varying receptive fields using two lightweight dilation branches and a gating function. Unlike ASPP or standard deformable convolution, it adapts the effective receptive field according to local content without introducing heavy computation. LCSF replaces the standard decoder with a linear semantic-guided cross-scale fusion that uses high-level features to refine low-level spatial details via channel- and spatial-attention. This preserves contours and suppresses background interference, outperforming standard lightweight decoders. As shown in Fig 1.

### 3.3 Overall network architecture design

#### 3.3.1 Lightweight backbone via re-parameterization.

To satisfy the dual requirements of segmentation accuracy and deployment efficiency, MobileNetV2 was adopted as the backbone and further enhanced with re-parameterized depthwise separable convolution (DS-Conv) blocks. Unlike conventional multi-branch designs that retain their training topology during inference and therefore introduce persistent computational and memory overhead, structural re-parameterization decouples training-time representational capacity from inference-time efficiency.

**Fig 2:**
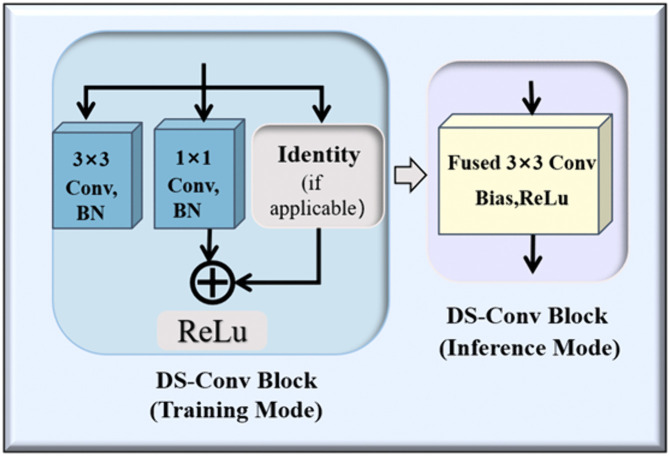
The re-parameterized DS-Conv block, illustrating the distinct topologies for training and inference phases.

As shown in Fig 2, illustrates DS-Conv block multi-branch fusion. During training, identity and convolution branches are used to enrich representation. During inference, branches are fused into a single layer to reduce computation without affecting feature quality.

#### 3.3.2 Scale-Adaptive efficient dynamic pyramid module.

**Fig 3:**
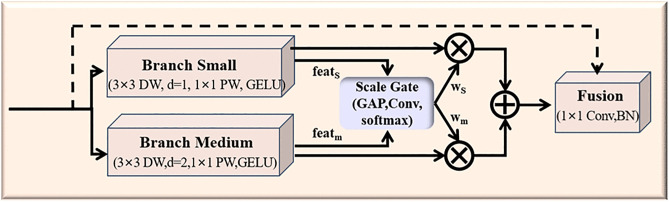
The architecture of the Scale-Adaptive Efficient Dynamic Pyramid module.

In alpine grazing scenes, yak targets exhibit substantial variation in scale and posture. SA-EDP uses two lightweight branches with different dilation rates and a gating function to dynamically adjust the receptive field for each spatial position. This allows adaptive geometric representation at low computational cost, outperforming fixed-scale ASPP or simple multi-scale fusion in capturing fine-grained local features. In contrast, deformable convolution can model geometric variation more flexibly, but its explicit offset learning introduces non-negligible computational overhead that is unfavorable for edge deployment. To provide a lightweight alternative, we design the Scale-Adaptive Efficient Dynamic Pyramid (SA-EDP) module. As shown in Fig 3.

Given an input feature map $\text{F}\in {\text{R}}^{\text{C}\times \text{H}\times \text{W}}$, the SA-EDP module employs two lightweight branches with different dilation rates, denoted by ${\text{d}}_{\text{1}}$ and ${\text{d}}_{\text{2}}$, to extract local-detail and broader-context features, respectively, yielding feature maps ${\text{f}}_{\text{1}}$ and ${\text{f}}_{\text{2}}$. Meanwhile, a lightweight gating function G predicts a spatial weight map $\text{M}\in [\text{0},\text{1}{]}^{\text{H}\times \text{W}}$:


$$\begin{array}{c} M=\sigma \left(Conv_{1\times 1}\left(ReLU\left(Conv_{3\times 3}\left(F\right)\right)\right)\right) \end{array}$$ (1)

where σ denotes the sigmoid activation function. The final output is obtained through element-wise interpolation between the two branches:


$$\begin{array}{c} Y_{i,j}=M_{i,j}\cdot f_{1\left(i,j\right)}+(1-M_{i,j})\cdot f_{2\left(i,j\right)} \end{array}$$ (2)

Crucially, from a theoretical perspective, this spatial interpolation mathematically constructs a continuous effective dilation rate (${\text{d}}_{\text{eff}}$) for each spatial coordinate (i,j):


$$\begin{array}{c} d_{eff}\left(i,j\right)=M_{i,j}\cdot d_{\text{1}}+(1-M_{i,j})\cdot d_{\text{2}} \end{array}$$ (3)

This formulation allows the receptive field to vary adaptively according to local content without introducing the latency associated with explicit offset regression. As a result, SA-EDP improves geometric adaptability while preserving the lightweight nature of the network.

#### 3.3.3 Linear cross-scale fusion module.

**Fig 4:**
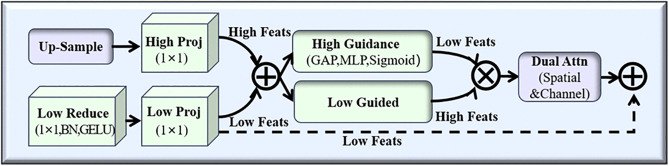
The architecture of the Linear Cross-Scale Fusion module.

In natural grazing environments, yak targets often present fine contour structures together with incomplete or degraded semantic cues caused by occlusion, distance, and background interference. Standard lightweight decoders often fail to preserve fine contours under occlusion or complex backgrounds. LCSF performs linear cross-scale semantic-guided fusion: high-level features generate channel-wise and spatial attention to refine low-level features, preserving contour continuity while maintaining efficiency. Ablation experiments (Table 2) show that LCSF improves mIoU from 92.00 to 92.04 with negligible additional cost, demonstrating empirical superiority over standard lightweight decoders. As shown in Fig 4.

**Table 1:** Training hyperparameter settings.

Hyper-parameter	Value
Frozen training epochs	10
Batch size (frozen stage)	8
Unfrozen training epochs	100
Batch size (unfrozen stage)	4
Input size	[512, 512]

**Table 2:** Ablation study results.

MobileNetV2	LCSF	SA-EDP	mIoU(%)	mPA(%)	Params	GFLOPs
			91.53	95.45	54.71	166.84
✔			92.12	96.81	5.81	52.87
✔	✔		92.93	96.62	1.91	9.23
✔	✔	✔	93.37	96.79	2.28	9.90

Let ${\text{F}}_{\text{high}}$ and ${\text{F}}_{\text{low}}$ denote the high-level semantic features and low-level spatial features, respectively. Initially, ${\text{F}}_{\text{high}}$ is upsampled to match the spatial resolution of ${\text{F}}_{\text{low}}$, yielding ${{\text{F}}^{\prime }}_{high}$, while $F_{low}$ undergoes channel reduction via a 1×1 convolution to yield ${{\text{F}}^{\prime }}_{low}$. To perform a directional “semantic-to-detail” information injection, ${F^{\prime }}_{high}$ is utilized to generate a channel-wise guidance $F_{mod}=G_{c}\left(\cdot \right)F_{low}^{\prime }$ gate ${\text{G}}_{\text{c}}\in [\text{0},\text{1}{]}^{\text{C}}$, which modulates the low-level features to suppress background noise:


$$\begin{array}{c} F_{mod}=G_{c}\left(\cdot \right)F_{low}^{\prime } \end{array}$$ (4)

where ⊙ denotes element-wise multiplication. The modulated low-level features and the high-level features are then aggregated:


$$\begin{array}{c} F_{att}=F_{high}^{\prime }+F_{mod} \end{array}$$ (5)

To further enhance the discriminability of the yak's main body and boundaries, a dual-attention mechanism is applied. Let ${\text{A}}_{\text{c}}$ be the channel attention weights derived from a Squeeze-and-Excitation (SE) block, and ${\text{A}}_{\text{s}}$ be the spatial attention weights generated from a depthwise separable convolutional block. The refined feature ${\text{F}}_{\text{att}}^{\prime }$ is computed as:


$$\begin{array}{c} F_{att}^{\prime }=A_{c}⊙\left(A_{s}⊙F_{att}\right) \end{array}$$ (6)

Finally, to preserve geometric stability and prevent the loss of fine-grained contours during the attention process, a residual connection re-injects the aligned low-level features:


$$\begin{array}{c} F_{out}=F_{low}^{\prime }+F_{att}^{\prime } \end{array}$$ (7)

By using high-level semantics to guide the reconstruction of low-level spatial details, LCSF improves contour continuity and suppresses background interference with limited computational cost, making it more suitable than a standard decoder for edge-oriented livestock segmentation.

## 4. Experimental platform and evaluation metrics

### 4.1 Environment configuration

All experiments were conducted on a unified platform equipped with an NVIDIA GeForce RTX 5060Ti GPU (16 GB memory), Windows 11, Python 3.12, and PyTorch with CUDA acceleration. All model weights were randomly initialized without any pretrained weights. The SGD optimizer was employed with momentum of 0.9 and weight decay of 1 × 10 ⁻ ⁴. The initial learning rate was set to 7 × 10⁻^3^ and decayed following a cosine annealing schedule to a minimum of 7 × 10 ⁻ ⁵. A combination of Dice loss and cross-entropy loss was adopted as the training objective to address foreground-background class imbalance. Mixed precision (FP16) training was enabled to reduce memory consumption. The main training hyperparameters are listed in Table 1.

### 4.2 Evaluation metrics

To quantitatively evaluate the segmentation performance of SALC-Net, four widely used metrics were adopted, including Pixel Accuracy (PA), Mean Pixel Accuracy (mPA), Intersection over Union (IoU), and Mean Intersection over Union (mIoU). These metrics are computed from the confusion matrix, where True Positive (TP) denotes yak pixels correctly classified as yak, False Positive (FP) denotes background pixels incorrectly classified as yak, True Negative (TN) denotes background pixels correctly classified as background, and False Negative (FN) denotes yak pixels incorrectly classified as background. The corresponding evaluation metrics are defined as follows:


$$\begin{array}{c} \text{PA}=\frac{\sum_{i}TP_{i}}{\sum_{i}n_{i}} \end{array}$$ (10)

$$\begin{array}{c} \text{mPA}=\frac{\text{1}}{K}\sum_{i=1}^{K}\frac{TP_{i}}{TP_{i}+FN_{i}} \end{array}$$ (11)

$$\begin{array}{c} {\text{IoU}}_{i}=\frac{TP_{i}}{TP_{i}+FP_{i}+FN_{i}} \end{array}$$ (12)

$$\begin{array}{c} \text{mIoU}=\frac{\text{1}}{K}\sum_{i=1}^{K}{\text{IoU}}_{i} \end{array}$$ (13)

where *K* denotes the number of classes. These metrics jointly reflect overall prediction accuracy, class-level consistency, and segmentation overlap quality.

## 5. Results and analysis

### 5.1 Ablation study for architectural components

Table 2 reports the ablation results of different architectural components. Each configuration was repeated three times with independent random seeds, and the mIoU values are reported as mean ± standard deviation to provide statistical support. Compared with the standard baseline, replacing the original backbone with MobileNetV2 substantially reduced model complexity, while also improving segmentation accuracy to 92.00 ± 0.18% mIoU. This result indicates that the lightweight backbone provides a more favorable balance between feature extraction efficiency and segmentation performance. After introducing the LCSF module, the parameter count and computational cost were further reduced from 5.813 M to 1.91 M and from 52.87 to 9.23 GFLOPs, respectively, while mIoU increased to 92.04 ± 0.29%. This reduction is mainly because LCSF replaces the parameter-heavy standard decoder head with a lightweight semantic-guided cross-scale fusion design. Although mPA showed a slight decrease at this stage, the gain in mIoU together with the substantial reduction in complexity suggests that LCSF is effective in improving contour-related segmentation quality under strict efficiency constraints. Finally, adding the SA-EDP module further improved the complete model to 93.14 ± 0.24% mIoU and 96.79% mPA, with only a small increase in parameters and GFLOPs. Relative to the standard baseline, the final SALC-Net reduced the parameter count and computational cost by approximately 95.8% and 94.1%, respectively. These results suggest that the combination of the lightweight backbone, LCSF, and SA-EDP provides a favorable accuracy-efficiency trade-off for yak segmentation on resource-constrained devices.

### 5.2 Comparative analysis with state-of-the-art models

The segmentation performance of SALC-Net was compared with several representative segmentation networks, and the quantitative results are reported in Table 3. Some high-capacity models, such as TransUNet, achieved relatively strong segmentation accuracy but exhibited limited inference speed because of their large model size and computational cost, which restricts their applicability in real-time edge deployment. In contrast, highly efficient models such as BiSeNet achieved very high throughput, but their segmentation accuracy was relatively limited for tasks requiring fine contour delineation. Similarly, DeepLabV3 + provided competitive segmentation accuracy, but at substantially higher computational cost.

**Table 3:** Performance comparison with other segmentation models.

Model	mIoU(%)	mPA(%)	Accuracy (%)	Params	GFLOPs	FPS
BiSeNet [29]	88.63	93.43	95.44	13.39	15.24	228
PSPNet [24]	88.68	93.49	95.53	46.71	118.43	85
PIDNet [30]	89.87	94.94	94.26	7.72	6.34	114
HRNet [25]	90.15	95.05	96.08	9.64	32.80	38
SegNet [21]	90.79	95.27	96.41	29.44	160.68	37
TransUNet [22]	91.03	95.19	95.19	105.91	129.29	18
DeepLabV3+ [23]	91.53	95.45	96.69	54.71	166.94	51
SALC-Net	93.37	96.79	97.42	2.28	9.90	129

By comparison, SALC-Net achieved the highest mIoU (93.37%) among all tested models, while maintaining a low parameter count (2.28 M), low computational complexity (9.90 GFLOPs), and real-time inference speed (129 FPS). These results suggest that SALC-Net provides a more favorable balance between segmentation accuracy and deployment efficiency than the compared methods in the yak segmentation task. In particular, the model achieved better contour-sensitive segmentation quality than lightweight high-speed baselines, while avoiding the excessive cost associated with heavier architectures. The results were obtained by applying each model to the same 310-image validation set under identical training and inference conditions (same GPU, batch size, and input resolution).

**Fig 5:**
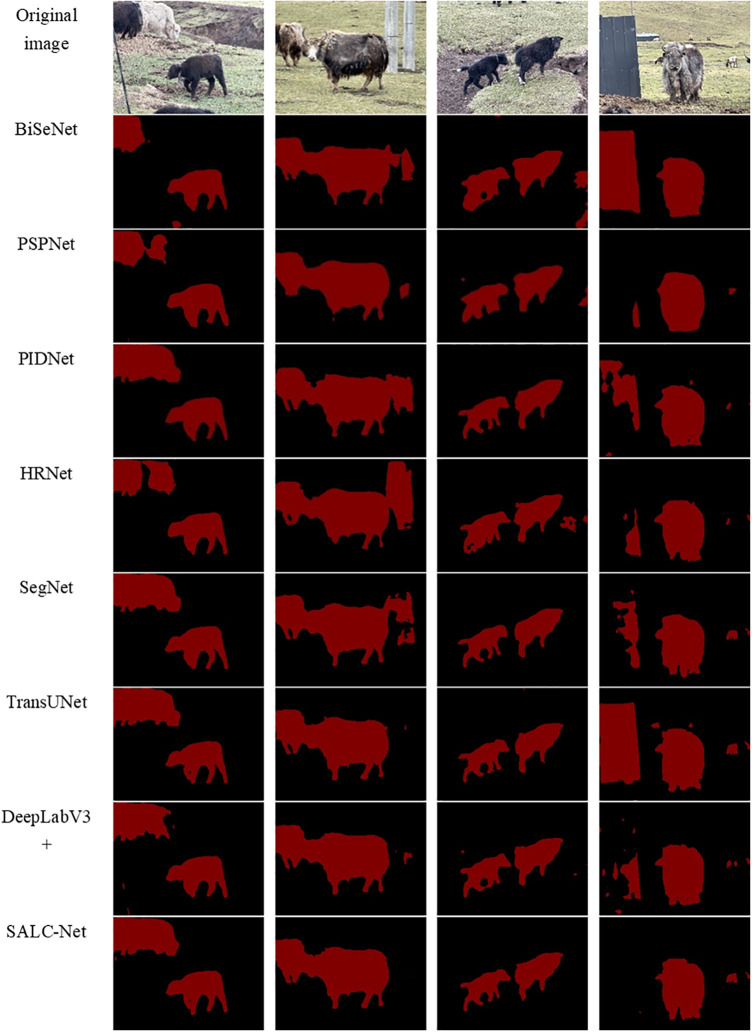
Qualitative comparison of segmentation results produced by different models.

To provide an intuitive assessment of the proposed SALC-Net, Fig 5 is referenced here to illustrate a comparative qualitative analysis against several state-of-the-art (SOTA) segmentation models, including BiSeNet, PSPNet, PIDNet, HRNet, SegNet, TransUNet, and DeepLabV3.

In the first column of Fig 5, SALC-Net produces significantly smoother and more refined contours compared with the “sawtooth” artifacts and jagged edges observed in other models, aligning more closely with the actual physical shapes of the yaks. In the second and fourth columns, where complex backgrounds such as utility poles or black iron fences frequently appear, SALC-Net demonstrates superior discriminative ability by suppressing background distractions and maintaining high segmentation purity.

The third column highlights SALC-Net’s capability to accurately capture fine-grained details of small-scale targets, such as ears and tails of yak calves, which are often fragmented in other models. Overall, this qualitative comparison demonstrates that SALC-Net overcomes limitations of background noise and information loss, producing more complete and contextually accurate segmentation results under complex pastoral conditions.

### 5.3 Cross-domain generalization capability

Table 4 summarizes the cross-dataset performance of SALC-Net and DeepLabV3+ on two additional datasets collected by our group: the Stellera chamaejasme dataset and the Crack dataset. Both datasets consist of natural highland scenes with complex textures, thin structures, and diverse object poses. SALC-Net achieved consistently higher mIoU and Accuracy than DeepLabV3+ across these datasets, indicating that the proposed architecture retains reasonable generalization ability beyond the primary yak segmentation dataset.

**Table 4:** Cross-dataset performance comparison.

Dataset Category	Model	mIoU(%)	mPA(%)	Accuracy (%)
Stellera chamaejasme dataset	DeepLabV3+	88.13	97.20	99.45
	SALC-Net	91.28	95.78	99.63
Crack dataset	DeepLabV3+	87.53	96.83	98.50
	SALC-Net	89.32	95.75	98.80

On the Stellera chamaejasme dataset, SALC-Net improved mIoU from 88.13% to 91.28% and Accuracy from 99.45% to 99.63%, suggesting better segmentation quality in natural scenes with complex textures. A similar trend was observed on the Crack dataset, where SALC-Net improved mIoU by 1.79% and Accuracy by 0.30%, demonstrating its effectiveness in capturing thin or irregular structures.

Overall, these results indicate that SALC-Net exhibits encouraging cross-dataset transferability, particularly for tasks that require effective boundary representation and structurally consistent predictions.

### 5.4 Generalization study analysis across challenging scenarios

To further evaluate the generalization and robustness of SALC-Net under realistic conditions, the dataset of 1,550 images collected from four highland regions was divided into four challenging scenario categories: complex background (542 images, 35%), occlusion/multi-target (388 images, 25%), pose/viewpoint variation (310 images, 20%), and illumination/weather variation (310 images, 20%). We acknowledge potential geographic and environmental biases, as the dataset only covers specific high-altitude grazing areas.

To mitigate overfitting and ensure consistency, we leveraged repeated ablation experiments and cross-dataset evaluations. Table 5 reports the segmentation performance (mIoU, mPA, Accuracy) for each scenario based on a single run. SALC-Net achieved the highest mIoU in the complex-background scenario (93.47%), demonstrating effective target-background discrimination. In the other three scenarios, mIoU remained above 92.70%, indicating stable performance under environmental disturbances such as target overlap, non-rigid pose changes, and illumination variation.

**Table 5:** Performance comparison across different challenging scenarios.

Category	Images	mIoU(%)	mPA(%)	Accuracy (%)
Complex background	542	93.47	97.01	97.65
Occlusion & multi-target	388	92.96	96.78	97.31
Pose &viewpoint	310	92.70	96.61	97.23
Illumination & weather	310	92.76	96.73	97.27

Overall, these results suggest that SALC-Net exhibits both robust and generalizable performance in challenging grazing environments, likely due to the complementary contributions of SA-EDP and LCSF in enhancing adaptive contextual representation and contour-preserving feature fusion.

## 6 Discussion

### 6.1 Architectural rationale and performance attribution

The results show that SALC-Net achieves a more favorable accuracy-efficiency trade-off than heavier segmentation models in unstructured grazing environments. This advantage appears to stem from the coordinated design of SA-EDP and LCSF rather than from model scale alone. In particular, SA-EDP improves the representation of scale variation and non-rigid deformation by providing lightweight dynamic receptive field adaptation, which is important for yak targets with diverse poses and body configurations. Meanwhile, LCSF enhances cross-scale feature fusion during decoding and helps preserve contour continuity under complex backgrounds, occlusion, and texture interference. The stable performance observed across challenging scenarios suggests that the proposed architecture is well-suited to livestock segmentation tasks requiring both boundary sensitivity and computational efficiency.

### 6.2 Limitations and practical implementation

Several limitations should be noted. First, the current framework is based on single-view RGB images and does not incorporate depth information, which limits the stability of absolute size estimation under varying distances and viewpoints. Second, although the cross-domain results indicate encouraging transferability, the model is still primarily optimized for yak segmentation in natural grazing scenes, and its generalization to broader domains requires further validation. Third, the dataset employed an 80:20 train-validation split without a separate held-out test set; the 310-image validation set was used for both model selection and final performance reporting, and future work with a larger dataset should employ a three-way partition to provide unbiased final evaluation. In this study, the validation set was also treated as a held-out test set for performance assessment.

## 7 Conclusion

This study presents SALC-Net, a lightweight segmentation framework designed to improve the trade-off between segmentation accuracy and computational efficiency for agricultural edge applications. By targeting the challenges posed by non-rigid livestock objects, the proposed architecture enhances feature representation for scale variation and pose deformation while maintaining low computational cost. Specifically, the re-parameterized backbone supports efficient inference, whereas the SA-EDP and LCSF modules improve adaptive context modeling and contour-preserving feature reconstruction, respectively.

Experimental results show that SALC-Net achieves a favorable balance between accuracy and efficiency, reaching 93.37% mIoU at 129 FPS. In addition, the preliminary deployment of a perception-to-analysis pipeline through a mobile application further suggests its practical potential for non-contact yak phenotyping in alpine pastoral environments. Nevertheless, limitations remain, particularly with respect to perspective-related variation under single-view RGB imaging. Future work will therefore investigate depth-aware sensing and low-bit model quantization to improve measurement robustness and energy efficiency for long-term deployment in resource-constrained environments.

## Supporting information

S1 Dataset (ZIP)

## References

[1] Shah AM, Bano I, Qazi IH, Matra M, Wanapat M. “The Yak”-A remarkable animal living in a harsh environment: An overview of its feeding, growth, production performance, and contribution to food security. Front Vet Sci. 2023;10:1086985. doi: 10.3389/fvets.2023.1086985 36814466 PMC9940766

[2] Jing X, Ding L, Zhou J, Huang X, Degen A, Long R. The adaptive strategies of yaks to live in the Asian highlands. Anim Nutr. 2022;9:249–58. doi: 10.1016/j.aninu.2022.02.002 35600551 PMC9092367

[3] Naz S, Chatha AMM, Ullah Q, Farooq M, Jamil T, Muner RD, et al. Genomic Adaptation, Environmental Challenges, and Sustainable Yak Husbandry in High-Altitude Pastoral Systems. Vet Sci. 2025;12(8):714. doi: 10.3390/vetsci12080714 40872665 PMC12390255

[4] Ma W, Qi X, Sun Y, Gao R, Ding L, Wang R, et al. Computer Vision-Based Measurement Techniques for Livestock Body Dimension and Weight: A Review. Agriculture. 2024;14(2):306. doi: 10.3390/agriculture14020306

[5] Yang SX, Han Y, Ma W, Tulpan D, Li J, Li J, et al. Review of computer vision for livestock body conformation assessment. Agric Commun. 2025;3:100099. doi: 10.1016/j.agrcom.2025.100099

[6] Menezes GL, Mazon G, Ferreira REP, Cabrera VE, Dorea JRR. Artificial intelligence for livestock: a narrative review of the applications of computer vision systems and large language models for animal farming. Anim Front. 2025;14(6):42–53. doi: 10.1093/af/vfae048 39764529 PMC11700613

[7] Guarnido-Lopez P, Pi Y, Tao J, Mendes EDM, Tedeschi LO. Computer vision algorithms to help decision-making in cattle production. Anim Front. 2025;14(6):11–22. doi: 10.1093/af/vfae028 39764526 PMC11700597

[8] Zhang Y, Sun Z, Zhang C, Yin S, Wang W, Song R. Body weight estimation of yak based on cloud edge computing. J Wireless Com Network. 2021;2021(1). doi: 10.1186/s13638-020-01879-y

[9] Peng Y, Peng Z, Zou H, Liu M, Hu R, Xiao J, et al. A dynamic individual method for yak heifer live body weight estimation using the YOLOv8 network and body parameter detection algorithm. J Dairy Sci. 2024;107(8):6178–91. doi: 10.3168/jds.2023-24065 38395405

[10] Xu B, Mao Y, Wang W, Chen G. Intelligent weight prediction of cows based on semantic segmentation and back propagation neural network. Front Artif Intell. 2024;7:1299169. doi: 10.3389/frai.2024.1299169 38348210 PMC10859394

[11] Yue C, Zhang X, Zhang Y, Jiang H, Miao H. Automatic and accurate measurement of cattle body based on a lightweight YOLOv8-Pose model and 3D point cloud. Mach Learn Appl. 2025;20:100662. doi: 10.1016/j.mlwa.2025.100662

[12] Chen X, Guo X, Li Y, Liu C. A Lightweight Automatic Cattle Body Measurement Method Based on Keypoint Detection. Symmetry. 2025;17(11):1926. doi: 10.3390/sym17111926

[13] Weng Z, Hao W, Gong C, Zheng Z. YOLOv8-DMC: Enabling Non-Contact 3D Cattle Body Measurement via Enhanced Keypoint Detection. Animals (Basel). 2025;15(18):2738. doi: 10.3390/ani15182738 41007983 PMC12466762

[14] Yang G, Qiao Y, Deng H, Shi JQ, Song H. One-stage keypoint detection network for end-to-end cow body measurement. Eng Appl Artif Intell. 2025;146:110333. doi: 10.1016/j.engappai.2025.110333

[15] Deng H, Yang G, Xu X, Hua Z, Liu J, Song H. Fusion of CREStereo and MobileViT-Pose for rapid measurement of cattle body size. Comput Electron Agric. 2025;232:110103. doi: 10.1016/j.compag.2025.110103

[16] Hou Z, Zhang Q, Zhang B, Zhang H, Huang L, Wang M. CattlePartNet: an identification approach for key region of body size and its application on body measurement of beef cattle. Comput Electron Agric. 2025;232:110013. doi: 10.1016/j.compag.2025.110013

[17] Yang Y, Liu M, Peng Z, Deng Y, Gu L, Peng Y. A real-time feeding behavior monitoring system for individual yak based on facial recognition model. PeerJ Comput Sci. 2024;10:e2427. doi: 10.7717/peerj-cs.2427 39650506 PMC11623189

[18] Yang Y, Deng Y, Li J, Liu M, Yao Y, Peng Z, et al. An Effective Yak Behavior Classification Model with Improved YOLO-Pose Network Using Yak Skeleton Key Points Images. Agriculture. 2024;14(10):1796. doi: 10.3390/agriculture14101796

[19] Li J, Yang Y, Yao Y, Zou H, Guo X, Xiao J, et al. A dynamic yak heifer pose estimation model based on keypoint detection for complex environmental monitoring. J Dairy Sci. 2025;108(12):13704–19. doi: 10.3168/jds.2025-27099 41043709

[20] Xie E, Wang W, Yu Z, Anandkumar A, Alvarez JM, Luo P. SegFormer: Simple and efficient design for semantic segmentation with transformers. In: Ranzato M, Beygelzimer A, Dauphin Y, Liang P, Vaughan JW, editors. Advances in Neural Information Processing Systems 34. 2021. p. 12077–90.

[21] Badrinarayanan V, Kendall A, Cipolla R. SegNet: A Deep Convolutional Encoder-Decoder Architecture for Image Segmentation. IEEE Trans Pattern Anal Mach Intell. 2017;39(12):2481–95. doi: 10.1109/TPAMI.2016.2644615 28060704

[22] Chen J, Lu Y, Yu Q, Luo X, Adeli E, Wang Y, et al. TransUNet: Transformers make strong encoders for medical image segmentation. arXiv. 2021. Available from: https://arxiv.org/abs/2102.04306

[23] Chen LC, Zhu Y, Papandreou G, Schroff F, Adam H. Encoder-decoder with atrous separable convolution for semantic image segmentation. In: Ferrari V, Hebert M, Sminchisescu C, Weiss Y, editors. Computer Vision – ECCV 2018. Cham: Springer; 2018. p. 833–851.

[24] Zhao H, Shi J, Qi X, Wang X, Jia J. Pyramid Scene Parsing Network. In: 2017 IEEE Conference on Computer Vision and Pattern Recognition (CVPR). 2017. p. 2881–90.

[25] Sun K, Zhao Y, Jiang B, Cheng T, Xiao B, Liu D, et al. High-resolution representations for labeling pixels and regions. arXiv. 2019. Available from: https://arxiv.org/abs/1904.04514

[26] Zhou G, Ye W, Li S, Zhao J, Wang Z, Li G, et al. FGPointKAN++ point cloud segmentation and adaptive key cutting plane recognition for cow body size measurement. Artif Intell Agric. 2025;15:783–801. doi: 10.1016/j.aiia.2025.06.003

[27] Kirillov A, Mintun E, Ravi N, Mao H, Rolland C, Gustafson L, et al. Segment anything. In: Proceedings of the IEEE/CVF International Conference on Computer Vision (ICCV). 2023. p. 4015–26.

[28] Wang J, Gou C, Wu Q, Feng H, Han J, Ding E, et al. RTFormer: Efficient design for real-time semantic segmentation with transformer. In: Koyejo S, Mohamed S, Agarwal A, Belgrave D, Cho K, Oh A, editors. Advances in Neural Information Processing Systems 35. 2022. p. 7423–36.

[29] Yu C, Wang J, Peng C, Gao C, Yu G, Sang N. BiSeNet: Bilateral segmentation network for real-time semantic segmentation. In: Ferrari V, Hebert M, Sminchisescu C, Weiss Y, editors. Computer Vision – ECCV 2018. Cham: Springer; 2018. p. 334–49.

[30] Xu J, Xiong Z, Bhattacharyya SP. PIDNet: a real-time semantic segmentation network inspired by PID controllers. In: Proceedings of the IEEE/CVF Conference on Computer Vision and Pattern Recognition (CVPR). 2023. p. 19529–39.

[31] Xiao X, Zhao Y, Zhang F, Luo B, Yu L, Chen B, et al. BASeg: Boundary aware semantic segmentation for autonomous driving. Neural Netw. 2023;157:460–70. doi: 10.1016/j.neunet.2022.10.034 36434954

[32] Li H, Cai J, Liu T, Xiao Y, Liu C, Zhou C. UST-YOLO11Pose-TRM: An Attention-Enhanced Keypoint Detection and Transformer Regression Framework for Yak Body Measurement. Animals (Basel). 2026;16(10):1493. doi: 10.3390/ani16101493 42193783 PMC13203451

[33] Sandler M, Howard A, Zhu M, Zhmoginov A, Chen L-C. MobileNetV2: Inverted Residuals and Linear Bottlenecks. In: Proceedings of the IEEE/CVF Conference on Computer Vision and Pattern Recognition (CVPR). 2018. p. 4510–20.

[34] Chen Z, Cao A, Deng H, Mi X, Yang J. Accurate contour preservation for semantic segmentation by mitigating the impact of pseudo-boundaries. Int J Appl Earth Obs Geoinf. 2024;126:103615. doi: 10.1016/j.jag.2023.103615

[35] Feng T, Guo Y, Huang X, Qiao Y. Cattle Target Segmentation Method in Multi-Scenes Using Improved DeepLabV3+ Method. Animals (Basel). 2023;13(15):2521. doi: 10.3390/ani13152521 37570328 PMC10417518

[36] Zhao H, Mao R, Li M, Li B, Wang M. SheepInst: A High-Performance Instance Segmentation of Sheep Images Based on Deep Learning. Animals (Basel). 2023;13(8):1338. doi: 10.3390/ani13081338 37106902 PMC10134975

